# Quantifying Patterns of Change in Marine Ecosystem Response to Multiple Pressures

**DOI:** 10.1371/journal.pone.0119922

**Published:** 2015-03-17

**Authors:** Scott I. Large, Gavin Fay, Kevin D. Friedland, Jason S. Link

**Affiliations:** 1 NOAA-Fisheries, Woods Hole, Massachusetts, United States of America; 2 NOAA-Fisheries, Narragansett, Rhode Island, United States of America; Università di Genova, ITALY

## Abstract

The ability to understand and ultimately predict ecosystem response to multiple pressures is paramount to successfully implement ecosystem-based management. Thresholds shifts and nonlinear patterns in ecosystem responses can be used to determine reference points that identify levels of a pressure that may drastically alter ecosystem status, which can inform management action. However, quantifying ecosystem reference points has proven elusive due in large part to the multi-dimensional nature of both ecosystem pressures and ecosystem responses. We used ecological indicators, synthetic measures of ecosystem status and functioning, to enumerate important ecosystem attributes and to reduce the complexity of the Northeast Shelf Large Marine Ecosystem (NES LME). Random forests were used to quantify the importance of four environmental and four anthropogenic pressure variables to the value of ecological indicators, and to quantify shifts in aggregate ecological indicator response along pressure gradients. Anthropogenic pressure variables were critical defining features and were able to predict an average of 8-13% (up to 25-66% for individual ecological indicators) of the variation in ecological indicator values, whereas environmental pressures were able to predict an average of 1-5 % (up to 9-26% for individual ecological indicators) of ecological indicator variation. Each pressure variable predicted a different suite of ecological indicator’s variation and the shapes of ecological indicator responses along pressure gradients were generally nonlinear. Threshold shifts in ecosystem response to exploitation, the most important pressure variable, occurred when commercial landings were 20 and 60% of total surveyed biomass. Although present, threshold shifts in ecosystem response to environmental pressures were much less important, which suggests that anthropogenic pressures have significantly altered the ecosystem structure and functioning of the NES LME. Gradient response curves provide ecologically informed transformations of pressure variables to explain patterns of ecosystem structure and functioning. By concurrently identifying thresholds for a suite of ecological indicator responses to multiple pressures, we demonstrate that ecosystem reference points can be evaluated and used to support ecosystem-based management.

## Introduction

Natural environmental variation [1–3] and multiple human uses, such as coastal-zone development and the harvest of living marine resources (LMRs; [4–6]), influence the structure and function of marine ecosystems. Ultimately, these pressures can also have direct implications on management actions designed to conserve these systems [7]. Ecosystem-based management is a holistic living resource management approach that concurrently addresses multiple human uses and ecosystem processes [7,8]. Similar to any complex system of decision-making, EBM requires the development of goals, relevant ecosystem assessments, and decision criteria for management action [9,10].

A specific ocean-use sector where EBM has been repeatedly explored is ecosystem-based fisheries management (EBFM). EBFM seeks to identify, among multiple pressures and responses, the suite of management options that best address the myriad tradeoffs for fisheries within an ecosystem [11]. Fish and fisheries are known to respond to a triad of pressures, including human-induced, internal system dynamics, and natural environmental variability [12,13]. A key challenge hindering fuller implementation of EBFM has been the delineation of appropriate decision criteria cognizant of these multiple pressures and responses [14,15]. Development of ecosystem-based thresholds has high utility.

Ecosystem thresholds occur when a small change in a pressure causes either a large response or an abrupt change in the direction of ecosystem state or function [12,16–18]. Using ecological indicators of ecosystem status, pressure-response thresholds (i.e., single-indicator response to a single-pressure) can be calculated from known functional forms such as piecewise regression models or estimated with generalized additive models [19,20]. An understanding of how single indicators respond to single pressures can be used to inform decision criteria for management action, however, multiple pressures often influence ecosystems simultaneously. Pressure-response thresholds can be extended into two-covariate thresholds (i.e., single-indicator response to two pressures) that can be used to identify how multiple pressures influence indicator values [21], however, reconciling multiple pressure-response thresholds from a suite of indicators may not be feasible. Here, we introduce a multivariate technique to identify thresholds of response to multiple pressures, for a suite of ecological indicators.

Gradient forest analysis [22] extends random forest [23], which fits an ensemble of regression tree models between ecological indicators and environmental and anthropogenic variables. Gradient forest accumulates standardized measures of indicator changes along the gradients for multiple indicators and uses them to build cumulative response curves, which are empirical nonlinear functions of change for each pressure variable. When considered as a multivariate ordination method, the cumulative response curve for each pressure variable can be expressed as a vector on a scree-plot, where vector length is determined by the importance of each ecologically informed transformation of the pressure variables. Gradient forest has been used to quantify spatial gradients in species distributional response to environmental variables for use with marine spatial planning [22–25]. Here, we present the first application of gradient forest exploring marine ecosystem response to anthropogenic and environmental pressures. The overall goal is to compare and contrast ecosystem response to multiple pressures and identify ecosystem thresholds to facilitate development of ecosystem reference points for ecosystem-based fisheries management [14,15]. Gradient forest enables us to explore the empirical shape and magnitude of changes in composition of ecosystem response along environmental and anthropogenic pressure gradients, and to identify critical values along these gradients that correspond to threshold changes in composition.

## Materials and Methods

### Ecological indicators

The ecological indicator data used in this study were compiled from Northeast Fisheries Science Center (NEFSC) bottom trawl surveys (BTS) that provide information regarding the regional ecology and oceanography of the NES LME, which spans the continental shelf from Cape Hatteras to Nova Scotia (data source: http://iobis.org/mapper/?resource_id=1435). These data are routinely used to monitor trends in abundance and distribution of important living marine resources for the region [26–28]. Using a random depth stratified survey design, the NEFSC scientific monitoring program has sampled 350–400 stations throughout the NES LME biannually since 1963. Stations within each depth-region stratum were randomly sampled and surveyed fish and invertebrates were sorted according to species, weighed, and the length was measured. Survey data were aggregated spatially and averaged across seasons to create annual averages for each ecological indicator from 1964–2010 [29]. We chose a suite of six ecological indicators that have been suggested and vetted as useful indicators for assessing ecosystem status and function for the NES LME [15,30–32]: overall fish community length, planktivore and benthivore to piscivore and shrimp-fish feeder ratio, pelagic to demersal ratio, species richness, indicator species (Longhorn sculpin, *Myoxocephalus octodecemspinosus*), and trophic level (Table 1).

**Table 1:** Ecological indicators used as response variables in gradient forest analysis.

Indicator	Abbreviation	Definition	Indicator rationale
Length (mean)	length_mean	mean length (cm) of individual fish for all spp. From BTS [15,29,47–50]	size distribution
Planktivore and benthivore to piscivore and shrimp-fish feeder ratio (index)	plank	ratio of abundance of lower trophic level guilds to upper trophic level guilds from the BTS [15,31,32]	trophic dynamics, energy flow, community structure
Pelagic to demersal ratio (index)	pd_ratio	ratio of abundance of pelagic and demersal fishes from BTS [15,31,32]	energy flow, community structure
Species richness (frequency)	richness	number of surveyed spp. from BTS [47,51–53]	aggregate community status
Longhorn sculpin biomass (t)	b_scul	total biomass of longhorn sculpin from BTS [46]	sensitive species, index of disturbance
Trophic level (mean)	TL_mean	mean trophic level of surveyed spp. weighted by abundance (biomass) [4,47,54,55]	how energy flow within an ecosystem is processed and distributed

### Pressure variables

We chose environmental pressure variables that influence ecosystem circulation patterns, primary production, vertical mixing, and the availability of nutrients across multiple geographic and temporal scales (Table 2). Fishing pressure has proven a challenge to estimate from an ecosystem perspective and the scientific community has yet to reach consensus on best practices for its measure [33–36]. We quantified fishing pressure using two variables: total commercial landings and exploitation. Landings, the total live weight of commercial species landed in the NES LME, serves as an estimate of removals from the system and is calculated by aggregating data reported to the National Marine Fisheries Service (NMFS) by dealers at weigh-out, logbooks, and vessel trip reports [28,37]. Fishing pressure is influenced by both fishing effort and resource potential [33,34], so we also quantified fishing pressure as exploitation: the ratio landings to surveyed biomass [35]. However, ecological indicators are also formulated with survey data including surveyed biomass, which may result in problems with confounding. Beyond influencing the current ecosystem state, fishing pressure may also have lagged effects on ecosystems [38]. Therefore, we also introduced a one-year time lag for both measures of fishing pressure.

**Table 2:** Environmental factors used as pressure variables in gradient forest analysis.

Pressure	Definition	Pressure Rationale	Data source
Atlantic multidecadal oscillation (AMO)	Measure of basin-scale warm and cold phases in the Atlantic	Provides a measure of variance in thermohaline circulation patterns [56]	http://www.esrl.noaa.gov/psd/data/correlation/amon.us.data
Winter North Atlantic Oscillation (NAO_w)	Winter (December-March) Average of relative strength between subpolar (Icelandic) low and subtropical (Azores) High atmospheric pressure cells (index)	Influences temperature, precipitation, and wind fields [31,32]	S1_Data.csv
Sea Surface Temperature (SST)	Mean surface temperature of the NES LME waters (°C)	Species' distribution, growth, and many ecological and biological processes [57]	http://www.ncdc.noaa.gov/data-access/marineocean-data/extended-reconstructed-sea-surface-temperature-ersst-v3b
Precipitation (Precip)	Annual precipitation in the catchment areas associated with the NES LME (cm)	Influences oceanic conditions such as salinity, stratification, and nutrient supply [34,58]	http://cdiac.ornl.gov/ftp/ushcn_v2_monthly/ Monthly data partitioned to 1 degree boxes and assigned to the NES LME drainages

### Random forests

We applied random forest methods from the R package “randomForest” [39,40] to time series of ecological indicators to assess the importance of environmental and anthropogenic pressures on ecosystem structure and function. Random forests are composed of regression trees, where indicator values are partitioned into two groups at a specific split value *v* for each predictor *p* to maximize homogeneity [23]. At each partition, splits are selected to minimize the sum of squared deviations from the group mean, termed impurity. Partitions are recursively split until a partition becomes a terminal node. The importance of a split within each node in the tree is measured as the reduction in the amount of variation explained by the partition.

A random forest is an aggregation of the results from an ensemble of regression trees that synthesizes output with high classification accuracy and accounts for interactions among predictor variables [39,41]. An independent bootstrap sample of data (resampled with replacement) is used to fit each tree (0.632, on average). Each partition within a tree is then split on the best of a random subsample of predictor variables. Data not selected in the bootstrap sample, the out-of-bag (OOB) data, are used to provide cross validations of generalized error estimates. Three important metrics are used with random forests: the goodness-of-fit $R_{s}^{2}$ for indicator s, the importance *I*_*sp*_ of each predictor *p* (here the pressure variables), and the raw importance value *I*_*spvt*_ for a predictor at each split value *v* in each tree *t*. Predictor importance *I*_*sp*_ quantifies the contribution of a predictor to the model goodness-of-fit by computing the prediction error of the model without the predictor and comparing it to the prediction error of the full model. Specifically, *I*_*sp*_ is estimated as the increase in OOB mean square prediction error when the predictor is randomly permuted while the other variables in the model remain constant, effectively removing the predictor signal.

The proportion of variance explained in a random forest, or the goodness-of-fit $R_{s}^{2}$ indicator *s* is defined as

$$R_{s}^{2}\;=\;1-\sum_{i}\frac{{{(X}_{si}-{\hat{X}}_{si})}^{2}}{{\left(X_{si}-{\overset{-}{X}}_{s}\right)}^{2}}$$ (1)

Where *X*_*si*_ is the *i*th observation of indicator *s*, ${\hat{X}}_{si}$ is the OOB prediction, and ${\overset{-}{X}}_{s}$ is the mean value.

The goodness-of-fit $R_{s}^{2}$ for each random forest is portioned among predictor variables in proportion to their importance *I*_*sp*_, such that $R_{sp}^{2}$ (predictor *p* for indicator *s*) is calculated as

$$R_{sp}^{2}\;=\;\frac{R_{s}^{2}I_{sp}}{\sum pI_{sp}}$$ (2)

The importance of a predictor variable for the ecosystem $R_{p}^{2}$ is estimated by averaging $R_{sp}^{2}$ across all indicators, such that

$$R_{p}^{2}\;=\;\frac{1}{N}\sum_{s}R_{sp}^{2}$$ (3)

We used a conditional permutation approach to account for correlations between predictor variable values [42]. Values for each predictor were only permuted within data defined by splits on any other predictors that were correlated above a threshold of r = 0.5 [22].

### Gradient forests

Changes in ecosystem attributes along environmental and anthropogenic gradients were identified using gradient forest methods. Random forest methods are useful for quantifying the ability for pressure variables to predict response variables and the importance of each response variable to these predictions. Gradient forests integrate individual random forests analyses over many response variables, which are used to develop flexible, non-parametric functions to quantify thresholds in indicator response to anthropogenic and environmental pressures (see diagram and further discription of these methods in [22]).

For the NES LME, we developed data matrices for both pressure (year x pressure variable) and response (year x ecological indicator) variables. Threshold estimates along gradients of each environmental and anthropogenic pressure variable *p* were generated using the gradientForest package, which distributes *R* ^2^ values for all indicators among predictors in proportion to predictor importance *I*_*sp*_ and along the gradient of values for each predictor according to the density of raw importance values *I*_*spvt*_ ([22]; available online at: http://gradientforest.r-forge.r-project.org/]. The importance associated with a split value along a predictor gradient indicates the relative change in indicator value. Therefore, changes in indicator values are reflected in split importance. For each predictor *p*, the split values *v* and the importance values at each split *I*_*spvt*_ were combined from every tree in all random forests for each indicator *s*. For each indicator, importance values *I*_*spvt*_ were standardized by the density of observed values for each predictor *p* and normalized to sum to$\;R_{sp}^{2}$. A monotonic function proportional to the importance of splits with minimum 0 and maximum ${\;R}_{sp}^{2}$ was used to define the turnover *F*_sp_(*x*) for each indicator along a pressure gradient, which describes the cumulative shift in indicator value along each pressure gradient and provides an estimate of the importance for any given pressure value *x*. Cumulative ecosystem response *F*_p_(*x*) for each pressure variable was estimated as mean *F*_sp_(*x*) for all indicators. The cumulative ecosystem response *F*_p_ transforms ecological units reflecting changes in ecological indicator value for each pressure variable [22].

### Ordination of ecosystem response to pressure variables

Cumulative ecosystem response can be used to transform environmental and anthropogenic pressure data onto a common scale. Using principal component analysis, we used ordination to represent *F*_p_ transformed data as a biplot where coordinate position represents different patterns in ecological indicators, as associated with the pressure variables. Pressure variables were superimposed on the biplot as vectors indicating the direction and magnitude of the most important variables [22,24].

## RESULTS

### Model prediction performance for ecological indicators

Overall, ecological indicator mean prediction performance ($R_{s}^{2}$) of the random forest on the OOB samples was between 0 and 0.07 (Table 3). Ecological indicators that served as synthetic measures of energy flow (pelagic to demersal ratio, $R_{s}^{2}$ = 0.078; planktivore and benthivore to piscivore and shrimp-fish feeders ratio, $R_{s}^{2}$ = 0. 056), diversity (species richness, $R_{s}^{2}$ = 0.061), and a species sensitive to disturbance (longhorn sculpin biomass, $R_{s}^{2}$ = 0.07) had the highest mean ${\;R}_{s}^{2}$, suggesting that the anthropogenic and environmental pressures that we chose were able to predict variation in these measures. Size based indicators (mean length, $R_{s}^{2}$ = 0.037; trophic level, $R_{s}^{2}$ = 0.0) had the lowest mean prediction performance, which suggests that variation in these indicators was poorly predicted by the selected pressure variables. Trophic level was not predicted by any of the pressure variables and was eliminated from the gradient forest model.

**Table 3:** Mean and range of model performance by ecological indicator, $R_{s}^{2}$.

Ecological Indicator	$R_{s}^{2}$
Longhorn sculpin biomass	0.07 (0–0.21)
Length (mean)	0.037 (0–0.091)
Pelagic to demersal ratio	0.072 (0–0.14
Planktivore and benthivore to piscivore and shrimp-fish feeder ratio	0.056 (0–0.18)
Species richness	0.061 (0–0.12)
Trophic level (mean)	0 (0–0)

### Importance measures for pressure variables

**Fig 1:**
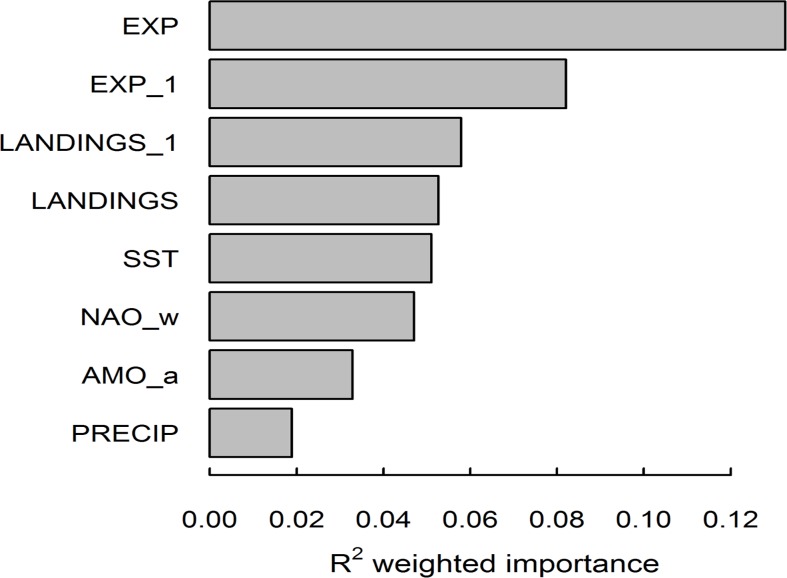
Importance of environmental and anthropogenic pressure variables weighted across ecological indicator outputs${\;R}_{sp}^{2}$. EXP, exploitation; EXP_1, 1-yr lagged exploitation; LANDINGS_1, 1-yr lagged landings (t); LANDINGS, landings (t); SST, sea surface temperature (°C); NAO_w, winter North Atlantic Oscillation Index; AMO_a, mean annual Atlantic multi-decadal oscillation index; PRECIP, precipitation.

The most important pressure variables for predicting ecological indicators were anthropogenic variables (Fig. 1). The mean importance of pressure variables ($R_{p}^{2}$), as measured by its contribution to prediction accuracy on the OOB response was between 0.019 and 0.13. Anthropogenic pressure variables (exploitation, $R_{p}^{2}$ = 0.13; 1-yr lagged exploitation, $R_{p}^{2}$ = 0.082; 1-yr lagged landings, $R_{p}^{2}$ = 0.058; landings, $R_{p}^{2}$ = 0.053) had higher mean ${\;R}_{p}^{2}$ than environmental variables, suggesting that these pressure variables were better able to predict variation in ecological indicators. Landings ($R_{p}^{2}$ = 0.053) were not as important as 1-yr lagged landings ($R_{p}^{2}$ = 0.058), however, exploitation ($R_{p}^{2}$ = 0.13) was more important than 1-yr lagged exploitation ($R_{p}^{2}$ = 0.082), therefore, including an estimate of community production available to harvest may be more useful in estimating ecosystem fishing pressure than by lagging pressure variables. Environmental pressure variables SST ($R_{p}^{2}$ = 0.051) and winter NAO ($R_{p}^{2}$ = 0.047) had importances comparable to landings, whereas AMO ($R_{p}^{2}$ = 0.033) and precipitation ($R_{p}^{2}$ = 0.019) had lower mean importance.

**Fig 2:**
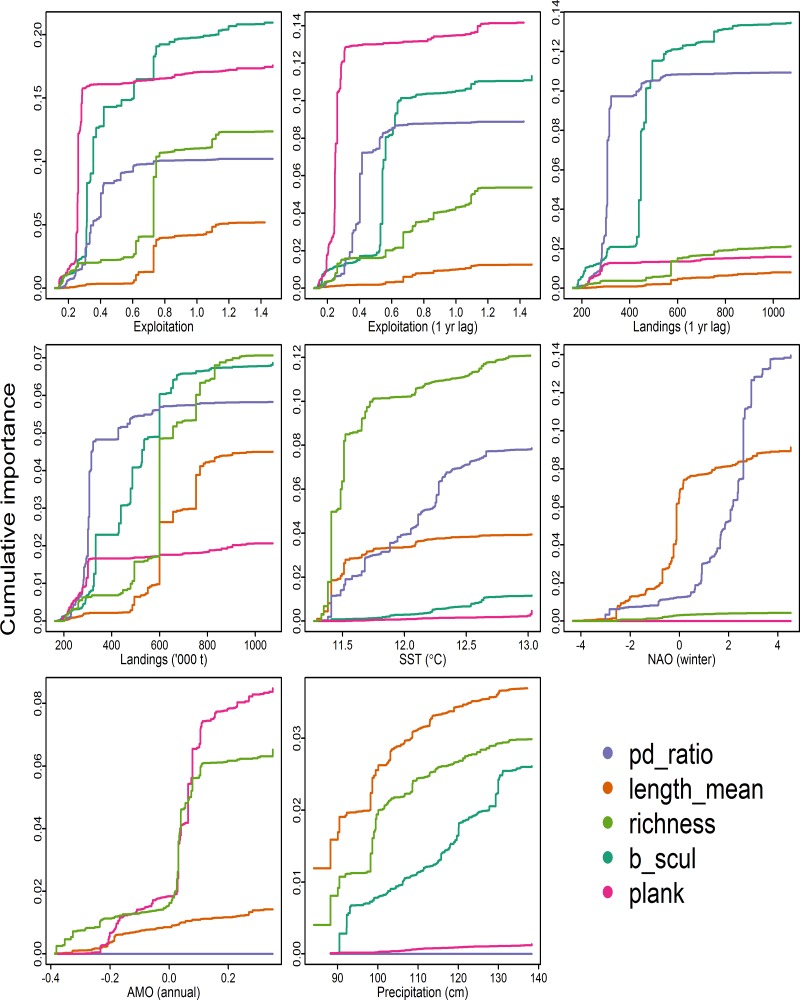
Cumulative shifts (in *R* ^2^ units) of ecological indicator value across the gradient of environmental and anthropogenic pressure variables. Each plot is scaled to the maximum cumulative response to allow for direct comparison of ecological indicator response to each pressure variable. Ecological indicator abbreviations are listed in Table 3.

Thresholds in ecological indicator response to pressure variables becomes evident when the cumulative predictive performance ($R_{s}^{2}$) of each ecological indicator is integrated along each pressure gradient (Fig. 2). Indicators of energy flow (planktivore and benthivore to piscivore and shrimp-fish feeders ratio, exploitation ~ 0.25; pelagic to demersal ratio, exploitation ~ 0.4) and a species sensitive to disturbance (Longhorn sculpin biomass, exploitation ~ 0.3) have a threshold response when exploitation is between 0.2 and 0.4, whereas indicators of diversity (species richness, exploitation ~ 0.8) and body size (length, exploitation ~ 0.8) have a threshold response when exploitation is ~ 0.8. These differing responses between types of ecological indicators suggests that diversity and body size may not be as sensitive to selected pressures as indicators of energy flow. Indicator threshold responses to 1-yr lagged exploitation were at levels similar to exploitation; however, the cumulative predictive performance ($R_{s}^{2}$) was generally lower. Pelagic to demersal ratio and longhorn sculpin biomass both had threshold responses when 1-yr lagged landings were ~300,000 t and 400,000 t. Indicators of energy flow (planktivore and benthivore to piscivore and shrimp-fish feeders ratio, pelagic to demersal ratio) and species sensitive to disturbance (Longhorn sculpin biomass) have a threshold response when landings were ~300,000 t, whereas indicators of diversity (species richness) and body size (length) have a threshold response when landings were ~ 600,000 t. Species richness had a distinct threshold response when SST ~ 11.5°C, whereas length and pelagic to demersal ratio had a gradual response with no distinct thresholds. Mean length and pelagic to demersal ratio had threshold responses to winter NAO when NAO ~ -1 and 2, respectively. Species richness and planktivore and benthivore to piscivore and shrimp-fish feeders ratio had a threshold response when AMO was slightly greater than 0. Mean length and species richness both had a threshold response when precipitation was ~ 100 cm, whereas longhorn sculpin biomass had a gradual response with increasing precipitation.

### Important breakpoints along pressure gradients

**Fig 3:**
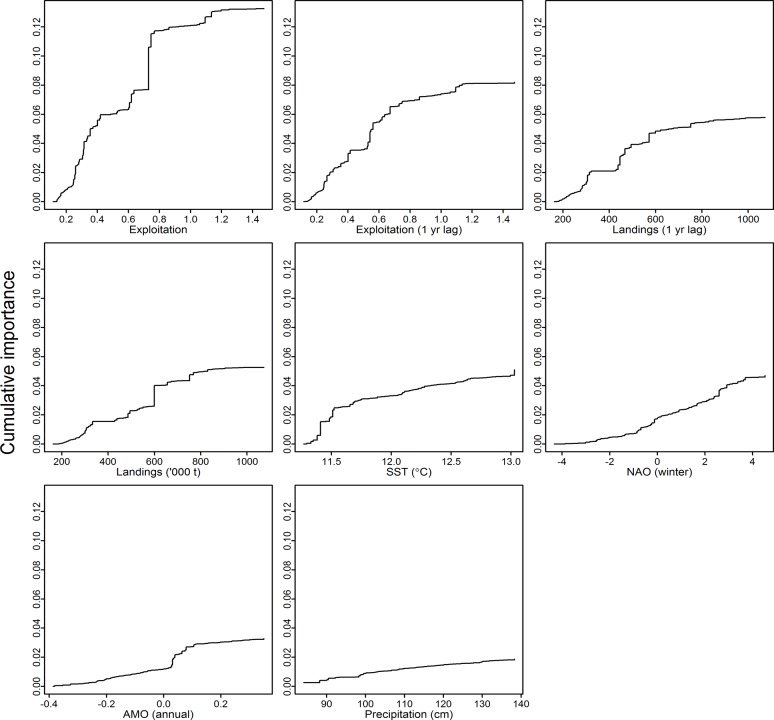
Cumulative shifts (in *R* ^2^ units) of aggregate ecological indicator response across the gradient for each environmental and anthropogenic pressure variable. Common scale allows for the direct comparison ecosystem response across pressure variables.

**Fig 4:**
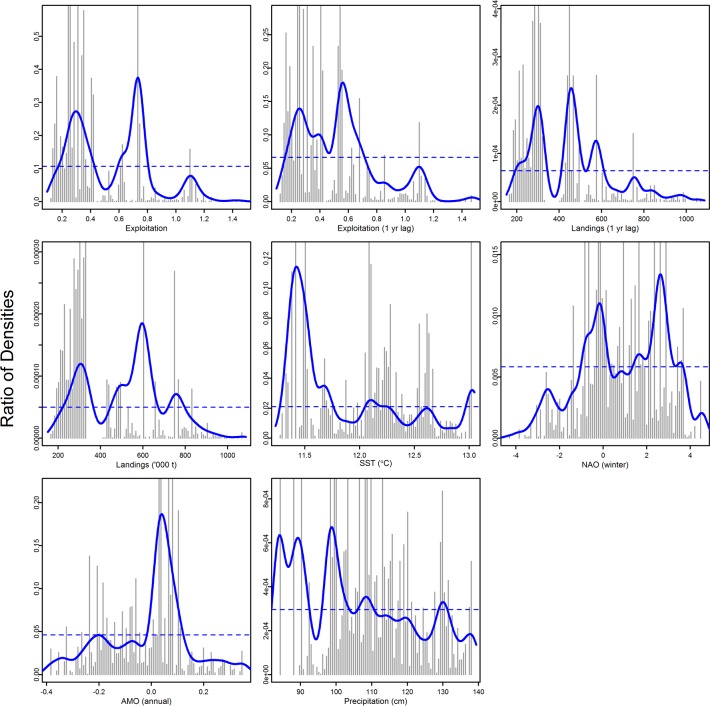
Threshold shifts in the value of aggregated ecological indicators along the gradient of environmental and anthropogenic pressure variables and reflect a rate of change in ecosystem processes. Binned raw importance of splits from random forests for ecological indicator value relative to the pressure variable on the horizontal axis. Density plots (lines) illustrate the estimated importance or turnover rate at any given pressure value, which is estimated as the ratio of the density of split importance to the density of observed predictor values along the predictor gradient. The dashed line indicates where the ratio is 1. Ratios > 1 indicate locations of relatively greater change in community composition, such that peaks in the density plot indicate threshold values for each environmental predictor where ecosystem status is expected to shift.

The weighted average of all ecological indicator responses (Fig. 3), or aggregate ecosystem response, for each pressure was calculated as the cumulative importance distributions of split improvement (Fig. 4) scaled by *R* ^2^ weighted importance (Fig. S1) and standardized by the density of observations (Fig. S2). Thresholds in ecosystem response are identified at peak values where the ratio is greater than one (Fig. 4). Important breakpoints in ecosystem composition are noted for exploitation (0.2, 0.6), 1-yr lagged exploitation (0.2), 1-yr lagged landings (220,000 t, 400,000 t, and 770,000 t), SST (11.0°C, 12.2°C, and 13.0°C), winter NAO (-1.5, 1.8), AMO (0.0), and precipitation (98 cm, 110 cm, and 130 cm).

### Biplot of ecological indicator values according to anthropogenic and environmental pressures

**Fig 5:**
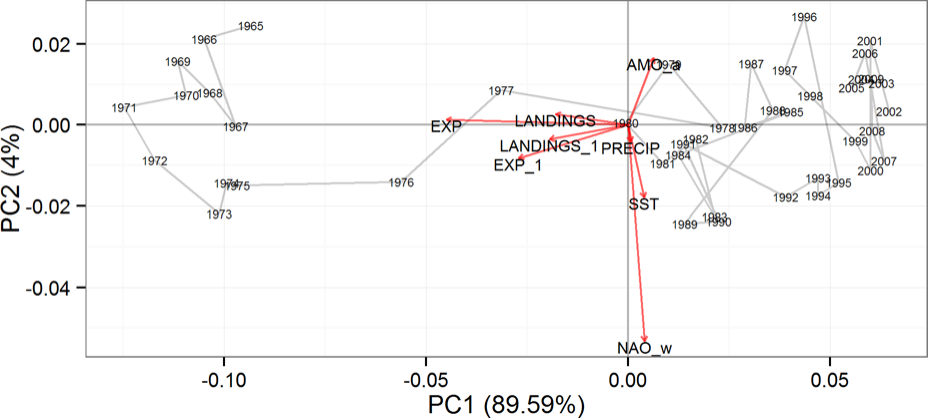
Biplot of the first two principal components display the coordinate positions and connecting segments for each year and indicating inferred compositional patterns. Environmental and anthropogenic pressure variables used in the analysis displayed as vectors.

Using cumulative importance functions (Fig. 3), shifts in ecological indicator value along pressure gradients can be mapped in multivariate space using principal component analysis (PCA; sensu Fig. 5 in [29]). The first two principal components account for 94.59% of the total variance. Coordinate position represents inferred ecosystem compositions, as associated with the anthropogenic and environmental pressure variables, as vectors. Each coordinate position is linked by a line segment to facilitate comparison as a time-series. Given the coordinate position of the inferred ecosystem compositions, two distinct groupings of data emerge: 1965–1977 and 1978–2010 (Fig. 5). Pressure variable vectors further indicate that exploitation, landings, and 1-yr lagged landings are important in defining this difference, which accounts for 89.5% of the explained variation. Both groupings vary according to the 2^nd^ PCA axis representative of environmental pressure variables, however, this axis only accounts for 4% of the explained variation.

## Discussion

In this study, we explored ecosystem response to a suite of anthropogenic and environmental pressure variables using an extension of random forest models, gradient forest. Specifically, we sought to 1) quantify the importance of environmental and anthropogenic pressure variables in predicting ecological indicator response, and 2) explore empirical shape and magnitude of changes in indicator response across these pressure gradients.

### Strengths and weaknesses of gradient forest approach

It can be useful to think of gradient forests in a multivariate sense, similar to PCA. The importance of each pressure variable is comparable to the length of a vector on a PCA scree-plot. But the gradient forest proves to be more informative, as the cumulative response curve is an ecologically informed transformation of the pressure variables, which can be used to predict and map potential patterns of ecological composition.

Given our objectives, gradient forest provided a robust and concise way to quantify the importance of pressure variables on ecological indicators of ecosystem status for the NES LME. We found anthropogenic pressure variables to be more important in predicting ecological indicator values than environmental variables. Although evidence suggests that the biological and ecological processes are being influenced by environmental pressures in the NES LME [43,44], the region has experienced far more significant pressure from fishing for centuries. Gradient forest also enabled us to identify threshold changes in ecosystem response to multiple pressure variables and to present these changes in a multivariate manner. Our results build upon previous findings exploring pressure-response relationships with similar ecological indicators and pressure variables for the NES LME using generalized additive models [20]. Large et al. [20] identified similar threshold levels and pressure response relationships for SST, AMO, and landings; however, each of these relationships were modeled as separate models and one main finding was the difficulty in synthesizing multiple pressures and multiple responses. The gradient forest approach we describe here provides a simple way to synthesize multiple indicators and multiple pressure variables using a common language, *R* ^2^ units, and across a common set of dimensions, via PCA. Further, the translation into a common language can also facilitate comparisons between multiple ecosystems to identify patterns in ecological indicator response to multiple pressures and levels of pressures that result in ecosystem thresholds. However, multiple pressures can also have non-additive [45] effects on ecosystems and additional exploration of thresholds between different combinations of pressures is warranted [21].

Compared to previous studies, model prediction performance on OOB sample values ($R_{s}^{2}$) from our study ($R_{s}^{2}$ range: 0–0.21) were notably low compared to Pitcher et al. ([24]; $R_{s}^{2}$ range 0–0.35) and Baker and Hallowed ([25]; $R_{s}^{2}$ range: 0–0.77). However, both Pitcher et al. [24] and Baker and Hallowed [25] were investigating community compositional changes across physical gradients and used species abundance metrics derived from survey data linked directly to physical variables. Here, we present aggregated ecological data for an entire large marine ecosystem linked to commercial landings and large-scale environmental variables. Ecosystems are highly dynamic and ecological data often contains more unexplained variability, however, gradient forest was still able to identify important variables and thresholds.

### Ecological indicators

Anthropogenic pressure variables were able to predict ecological indicator values better than environmental pressure variables. Further, threshold responses were more pronounced (i.e., more rapid increase on cumulative importance curves) for anthropogenic pressures compared to environmental pressures, and we identified levels of fishing pressure that resulted in a distinct ecosystem structure (Fig. 5). Care and context are important when interpreting indicator response to pressures, as magnitude and direction of ecosystem response to pressures may not be entirely straightforward. Here, we identify regions of change, both positive and negative, relative to pressure. Patterns of threshold responses were similar between exploitation and landings, suggesting that confounding between exploitation and ecological indicators did not influence our findings. However, as ecological indicators of ecosystem status have been developed, much of the impetus in indicator selection has been to measure the effects of fishing pressure on ecosystem attributes [35,36]. Given that EBFM is a management approach, the ability to control fishing pressure has been the rationale behind the use of ecological indicators that are heavily influenced by fishing pressure [15]. We suggest that ecological indicators should be reconsidered so they are relevant measures of ecosystem status and function without any pre-disposed sensitivity to any perceived pressure, thereby, creating a suite of ecological indicators that are more holistic and we can ensure that fishing and environmental thresholds can be identified. Given the gradient forest framework, additional ecological indicators could provide a much more comprehensive representation of how multiple pressures influence ecosystems without the fear of over-parameterizing models.

### Ecosystem thresholds

Using the gradient forest method, we found that increased fishing pressure (i.e., exploitation, landings, and both 1-yr lagged fishing pressure variables) resulted in rapid compositional change in ecological indicator values. Threshold responses in energy flow indicators occurred at lower levels of fishing pressure, suggesting that with increasing fishing pressure energy transfer within the NES LME is altered. As fishing pressure increases further, threshold responses in the diversity indicator occurs. Given the multiple community configurations that can produce similar diversity indices, Link [15] suggests that ecological indicators of diversity might be better suited as a precautionary tool. Perhaps the high level of fishing pressure that results in a diversity response might reinforce this suggestion. Longhorn sculpin, a scavenging generalist that rapidly increases in abundance in response to seafloor disturbance caused by bottom trawling [46], has drastic threshold responses that correspond to both types of fishing pressure.

Collectively our results indicate significant ecosystem responses when landings are at 400,000 t, which represent ~30% of the maximum ever observed for this system. Similarly, significant ecosystem responses are observed at exploitation rates of 20%. Certainly environmental pressures also elicit some response, yet fishing pressure is potentially the one variable that we can control. These results suggest that when landings approach 30% of those ever observed, or more practically that when the ratio of surveyed biomass to landings approaches 20%, the NES LME will likely be approaching an ecosystem threshold. These empirical derivations warrant further testing in other ecosystems to see if these approximate levels hold, but the method proposed here provides a highly feasible means to do so. Further, delayed effects of fishing and environmental pressures should be explored in more detail [38]. As we continue to move towards implementing EBM, the need for clearly defined decision criteria remains paramount. This gradient forest approach could serve as an important means for establishing ecosystem-level decision criteria to make EBM more of an operational reality.

## Supporting Information

S1 Data North Atlantic Oscillation index (December-March) accessed at https://climatedataguide.ucar.edu/climate-data/hurrell-north-atlantic-oscillation-nao-index-pc-based on 28 January 2015. (CSV)

S1 Fig Histogram and kernel density of splits location and importance. (TIF)

S2 Fig Histogram of splits location and importance, and kernel density of observations along the gradient. (TIF)
