# Supplementary figures and images for: Quantifying Patterns of Change in Marine Ecosystem Response to Multiple Pressures
Source: PLoS One. 2015 Mar 17;10(3):e0119922. doi: 10.1371/journal.pone.0119922 (PMC4362946; doi:10.1371/journal.pone.0119922)

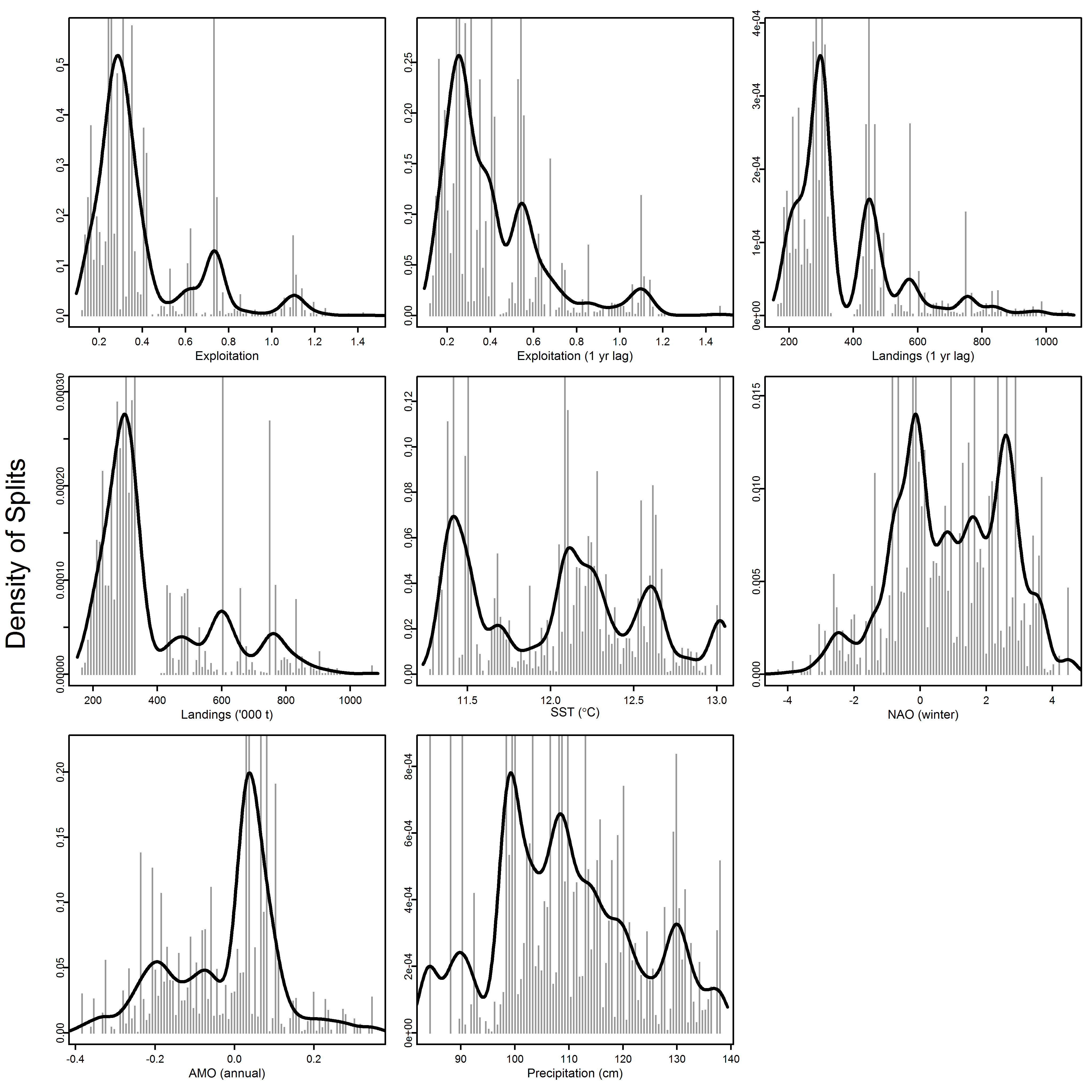

Supplement: S1 Fig — (TIF) [file pone.0119922.s002.tif]

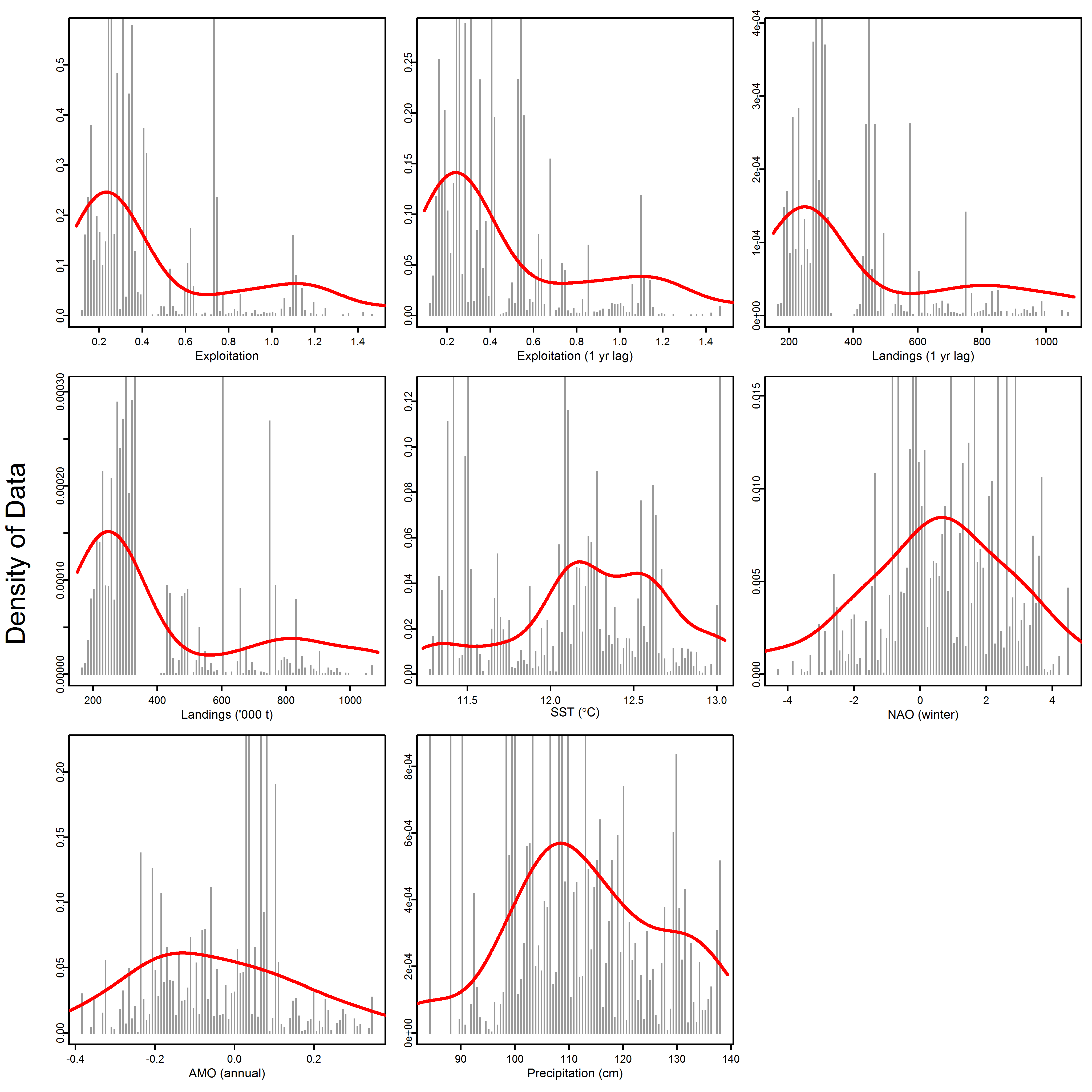

Supplement: S2 Fig — (TIF) [file pone.0119922.s003.tif]
